
JOURNAL INFORMATION
==============================
NLM Title Abbreviation: Med Chir Rev
NLM Journal ID: 0034617

The Medico-Chirurgical Review

Publisher: Biomedical Journal Digitization Project

ARTICLE INFORMATION
==============================
PMCID: PMC4966258
PMID: 29917629
Article version: 1
Subjects: Reviews, Edinburgh Medico-Chirurgical Transactions

On a New Method of Preserving Anatomical Preparations

Print publication date: 1828 Oct 1
Volume: 9
Issue: 18
First page: 404
Last page: 408
1828 Sep
License: This work is free of known copyright restrictions. For more information, please see PMC Back Issue Digitization.
License URL: https://creativecommons.org/publicdomain/mark/1.0/

==============================

the affected part, these not being observed until its size, or encroachment upon neighbouring parts, produces secondary attacks or alterations in contiguous textures, which rouses the attention of the patient. The exsanguine appearance of the patient, even at a very early period, and the uncommon depression of vital power which he experiences, would lead to the belief of a constitutional cause, either an alteration in the constituents of the blood, from which these diseased products are separated by the ordinary secreting power of vessels, or from a morbid alteration in the secreting powers themselves, or from both of these causes." 248.

Dr. Seymour appends a couple of cases where pain and vomiting, when they are attendants on this disease, " were effectually relieved for a considerable time by the administration of the prussic acid." Some very good plates are added to this paper, illustrative of the morbid structures above described.

---

### XIII.

*On a New Method of preserving Anatomical Preparations.* By JOHN DAVY, M.D. Physician to the Forces, &c.

[Ed. Med.-Chir. Trans. Vol. III.]

WE noticed some short time ago a " new mode of preserving dead subjects," by immersion in molasses—we have now to bring forward quite an opposite mode of preserving from corruption the " frail memorials"—not of man's existence, but of those maladies which have terminated that existence. Dr. Davy's zeal and research in the cause of medical science are very commendable, and we hope they will tend to benefit the profession and society at large. During the last two or three years, our author has been making trials of the method now to be described, and the success has exceeded his most sanguine expectations. Its advantages are triple—it is *cheap*—it is *durable*—and it clearly displays *minute structure.*

" Its great cheapness will be immediately obvious when I mention what it is, and that it is simply the sulphurous acid, which may be prepared in a manner equally economical and easy, by burning sulphur matches over water in any appropriate vessel,* agitating the water when the match ceases to burn, and, when the water is sufficiently impregnated with the acid gas, filtering the solution to render it clear and transparent." 231.

Of the durability of the process Dr. D. has no doubt. He has preparations by him which were made nearly three years ago, and nothing has been done to them all that time—no fluid has been added—no evaporation has taken place—and they are as perfect as when they were first immersed in the acid. They are merely confined in a bottle with a glass stopper, lubricated with a little cerate.

" From the experiments which I have made, the sulphurous acid appears to have nearly the same power in preventing the putrefaction of animal matter, as it has of stopping the fermentation of vegetable juices. I find that serum coagulated by this acid gas, and converted into a kind of jelly, may be kept in water several weeks,

---

* " The best kind of match for this purpose is that which is used in Italy,—cotton thread dipped in melted sulphur."

exposed to the air, without undergoing any change; that the fibrine of the blood thus treated is equally exempt from change, and that the red particles of the blood, rendered by it of the colour and consistence of thick tar, exposed in water to the air, become, indeed, covered with a kind of mildew or byssus, and emit a peculiar disagreeable smell, but not a putrid smell. I mention the serum, the fibrine, and the colouring matter of the blood, because they decompose and become putrid very readily (the two latter especially) sooner than most animal substances; and, therefore, what is applicable to them is *à fortiori* to anatomical preparations. I have made similar and direct trials of many preparations; I have exposed them to the air in this acid, and have taken them out of the acid and placed them in water exposed to the air, and the result has been satisfactory; shewing, that, after having been well acted on by the sulphurous acid, they are no longer liable to putrefaction. Some of these have undergone very little change during exposure to the air for several weeks in water; others have become soft and gelatinous, and have been partially reduced to a pulp, but without emitting any offensive odour, like that of putrid animal matter,—an odour rather like that of decaying vegetable matter affected with mildew." 232.

The third advantage consists in the display of minute structure—and this, perhaps, is the chief recommendation of the acid. It does not, like alum or alkohol, contract what is immersed in it—nor does it, like solutions of nitre and other salts, lose its transparency and become turbid. On the contrary, "it expands and developes the parts, some more some less, so as to magnify them and make them more distinct, effecting in structure what the lens does in vision; and, at the same time, it remains clear, so that the lens may still be employed to heighten the effect, and convey still more minute information of the object." This is almost miraculous, and we hope it is not exaggerated by the magnifying powers of the acid itself.

Dr. D. next enters into a detail of the effects of this preservative menstruum on the different textures. The following extract is interesting in many points of view.

"The skin immersed in the sulphurous acid swells considerably, and the cuticle is either thrown off or very easily detached. The cuticle of the sole of the foot, to mention a particular instance, is rendered almost transparent, and is but little thickened. When held between the eye and the light, its symmetrical structure is beautifully shewn, with innumerable dotted points, as it were, in the course of its linear wavings, which probably serve the purpose of pores.* If the cuticle, detached by means of sulphurous acid, is dried, it shews its peculiar structure still more distinctly and elegantly.

"The cutis, distended by the sulphurous acid, appears as a tissue of extremely minute fibres and particles, condensed towards the outer surface, when in contact with the cuticle, and loose internally, where it gradually blends itself with the adipose texture.

"Serous membranes, immersed in the acid, swell considerably, and lose very little of their transparency. The inner surface of a portion of pericardium, now before me, has an uneven surface, and exhibits when examined with a common lens, a slight appearance of pores; its section shews as if it were composed of layers, and its outer surface displays a loose intertexture of fine fibres. A portion of pleura, with the exception of the cut part, (which is very much thinner, and not visibly in layers,) appears very similar, but less distinctly.

* "Probably similar to the very minute cavities in the skin of the common toad, which allow light to pass through them, but not the most delicate probe, being covered with a very fine transparent membrane."

"On the cellular structure, the sulphurous acid has much the same effect as on the serous;—it distends it greatly, and preserves it transparent."

"Cartilage is not changed by the acid; neither the dense cartilages of the ribs, or the delicate ones attached to certain organs, as the tarsus, epiglottis, &c. It is equally inactive on bone. Nor does it appear to have any sensible effect either on muscular fibre, or on the substance of the brain and spinal chord, or of the nerves and ganglia. On mucous membranes its action is considerable: it does not appear to dissolve them in the slightest degree, but it distends them; renders them firm; and shews their peculiar structure in a very striking manner. As an example, I shall give a brief sketch of the primæ viæ. First, I may premise, that it displays the cuticle or epithelium, terminating abruptly in the œsophagus, just above the entrance of the latter into the stomach, and in the rectum, just within the circle of the anus, or the upper boundary of the sphincter muscle. In both, the termination is distinct, and abrupt, and unquestionable, as it appears in a preparation now before me.*

"When the epithelium is removed, (and it is most easily, either by placing it under a stream of water, or by the friction of the fingers,) and the mucous coat is brought distinctly into view, in the upper part of the œsophagus it appears very thin, provided with many and pretty large mucous follicles on each side, and several minute branches of nerves. With a magnifying glass of ordinary power, it seems to contain a most delicate tissue of white lines, running chiefly longitudinally, branching off slightly and anastomosing. The same appearance presents itself in the middle and lower part of the œsophagus, and more distinctly. But it is only in the upper part that any nerves can be seen distinctly ramifying in the semitransparent mucous tissue. In the middle no follicles are visible, and only a few near its termination. Where it terminates, there is, in one preparation before me, quite a zone of what appears to be minute follicles: But this does not shew itself in another preparation.

"At the cardiac orifice of the stomach many minute follicles present themselves. Where the gullet ends, and the stomach commences, there is a sudden change of the appearance of the mucous texture; the linear pencilling, (if I may be allowed the expression,) suddenly ends; and, as it were, a dotted one commences, and with some variations, extends throughout this organ. When magnified, it has the appearance of a very delicate lacework, formed in the upper part, by the close juxta-position of circular lines; and, in the lower, by lines in the form nearly of the figure of 8; and, in the great arch of the stomach, by lines irregularly tortuous. But this, it must be confessed, is not to be seen in every preparation that I have examined; and, therefore, I would rather limit myself by saying, that, in the stomach, the mucous texture has the appearance of a very delicate lacework of lines or vessels.

"At the commencement of the duodenum, the appearance of the texture again distinctly changes. Here it appears as if covered with tortuous threads or vessels twice or thrice the size of those of the mucous coat of the stomach; and, as the duodenum descends, the appearance strengthens. In some preparations there is an appearance of follicles, and of circular depressions but not in all I have examined; and, when seen, they are most distinctly seen with the naked eye. A longitudinal section, including the pylorus, strikingly and invariably shews a layer of a minute glandular structure, embracing and belonging to the mucous coat of the duodenum, which terminates abruptly at the pylorus, but gradually in the contrary direction, and disappearing, or nearly so, about an inch and a half from the pylorus. The same section, too, shews how very thick the muscular and cellular coats of the stomach are, in comparison with those of the duodenum.

* "It appears very questionable, that the stomach and intestines generally, the gall-bladder and the biliary ducts, the urinary, seminal, and the air passages, possess an epithelium analogous to cuticle and a continuation of it. All these parts must necessarily have a lining membrane, which seems to be their mucous coat, in each part somewhat different. It has been supposed that the mucus secreted by the membrane serves in lieu of cuticle, and it seems to be a probable conjecture."

"The inner coat of the jejunum, viewed with the naked eye, in sulphurous acid, appears to be distinctly villous, or as if studded with innumerable projecting capillary points; but this appearance vanishes when it is examined with a common lens; then, in some lights, it appears to be covered with convoluted threads, having a kind of centre, round which they are described, which to the unaided eye, seems a minute mucous gland; and, in other lights, as minute projecting laminæ variously bent.

"As the jejunum passes into the ilium, and as the ilium descends, another change takes place, and it is very strongly marked close to the termination of the latter intestine, where the mucous coat is truly villous;—so it appears to the naked eye in sulphurous acid; and, when examined in a favourable light with a lens, the villi exhibit the appearance of tubular projections rounded at the ends; and some of them have a form approaching to the conical, rather than the cylindrical. The sulphurous acid makes the glandular structure of the lower part of the ileum very distinct to the naked eye.

"This villous structure terminates suddenly at the valve of the colon (where the valve begins), and a new kind of structure succeeds it in the valve, and proceeds, with very little variation, of the same kind throughout the large intestines, even to their termination, not excepting the appendicula vermiformis. It resembles honey-comb in its appearance, more than any thing else to which I can compare it; but it is so very minute and delicate in every part of the large intestines, that it cannot be well distinguished, excepting with the aid of a magnifying glass. The glands, on the contrary, of the large intestines, appear very distinct in the sulphurous acid, and their orifices are easily seen." 240.

Dr. D. now proceeds to the more complicated structures, beginning with the nerves. We cannot give the following short passage in fewer words than the author has done.

"By rendering the fibrous neurilema, and the connecting cellular tissue, semi-transparent, and by expanding these textures, the sulphurous acid has a remarkable effect in displaying the nerves, with the exception of the ganglionic. It shews, almost without dissection, and still better when aided by dissection, which it facilitates, the parts of each fasciculus of nerves and their junctions; and, if the nerve be cut, the proportion of the medullary matter which it contains, which becomes projecting, squeezed out, owing to the pressure just alluded to. It shews very distinctly the fibrous nature of the optic nerves beyond their junction; but, at their junction, and towards their thalami, their substance appears to be exactly similar to the common medullary substance of the cerebrum. It shews the papillæ of the tongue to be each the termination of a minute nerve, and this without the aid of a magnifying glass. It demonstrates how, as the nerves proceed from their source, the proportion of medullary matter diminishes, and the thickness of their sheath increases; and this very remarkably in the nerves of the fingers. On the ganglionic nerves, judging from the very few trials which I have yet made, it appears to have but little effect, as if their composition were different from that of the other nerves, as has been lately maintained.

"It displays, too, almost without dissection, the composition of the vessels, and, I may add, of the passages and canals of the body generally; owing to its expanding, in different degrees, the several coats of which they consist." 243.

Dr. Davy thinks the sulphurous acid will prove equally useful in pathological as in simple anatomy. It expands most of the tissues, and has little solvent power on any of them—on coagulable lymph none at all.

"The specimens which I have by me, of diseased parts preserved in this acid, are satisfactory beyond my expectation; not so much as shewing the exact diseased appearance, such as it presented itself on dissection, as in giving a correct notion of the nature of the lesion, by magnifying and rendering more distinct the effects of the disease, contrary to what happens when the morbid parts are immersed in spirits of wine."* 245.

The sulphurous acid, Dr. D. observes, enables one to detect "the slightest *relaxation* of mucous membranes." We hardly know what the Doctor means by *relaxation*. When a man has got an inflamed *uvula*, he tells you the palate of his throat is "relaxed," or that it is "come down," but surely this is a very vague term to be used by a pathologist. The acid, Dr. D. also says, enables us to detect the cicatrices of old ulcers in the intestines. These cicatrices, according to his experience, seem to differ chiefly from sound mucous membrane, in containing no follicles, when the previous ulceration has been deep. When it has been superficial, it exhibits a structure more or less differing from sound membrane. He has in his possession a very delicate specimen of tubercles on a portion of pleura costalis, covered with a fine net-work of coagulable lymph, as distinct as when first immersed—and more distinct than before it was immersed in the acid. A portion of hepatised lung, thus preserved, shews the air-cells filled with an albuminous deposition, and the blood-vessels closed with the same.

"In all the specimens of aneurism which I have examined, and in every instance of tendency to it, I have found the inner and middle coat of the vessel diseased; the latter generally diminished in thickness, and rendered brittle, and sometimes entirely absorbed in the dilated part of a true aneurism, or in the part contiguous to the orifice communicating with the sac of the false; and the inner coat corresponding generally thickened, and corrugated, and easily broken. In many instances, since I have used the sulphurous acid, I have noticed a very strong tendency in the arteries to aneurism, with only incipient dilatation, in which the middle coat of the vessel has been reduced in parts to the thinness of the finest paper, and its colour changed yellow; and the inner membrane admitting of being easily detached from this, has been thickened to thrice or more its thickness in its sound state." 247.

The following precautions are necessary, in order to ensure success.

"1*st*, It is of consequence that the part to be preserved is immersed in the acid, as soon as possible; for if in the slightest degree putrid, it will not be well preserved, the fluid will become turbid, though frequently changed, and the preparation will not keep.

"2*dly*, If the part is putrid, as is often the case, when taken from the body, though the *post-mortem* examination is made a few hours after death, it should be immersed in a solution of chlorine in salt and water, till it is deprived of its putrid smell and tendency; then it may be washed clean, and put into the acid solution, without danger of its spoiling.

"3*dly*, Attention should be paid to the strength of the sulphurous solution, to proportion it to the nature of the part to be preserved, and the object in view in employing it. If the intention is to expand and develope the structure of the parts, for the purpose of demonstration, a strong solution answers best; if merely to preserve a part as little changed as possible, a weak solution is most successful, especially if cuticle, the visible epithelium or tendinous parts, are concerned, as a strong acid separates the former, and partially dissolves the latter." 248.

* "For the purpose of pathological research, the part to be examined may, with advantage, be immersed for a few hours in sulphurous acid, and then transferred to a cylindrical bottle, of thin and very clear glass, full of water, perfectly clear; very slight changes in the delicate structure of the part, before obscure, or not to be distinguished, will become apparent and distinct; and more particularly, very slight lesions and changes, whether recent or old, of the mucous membranes."
